# Foundry: a message-oriented, horizontally scalable ETL system for scientific data integration and enhancement

**DOI:** 10.1093/database/bay130

**Published:** 2018-12-17

**Authors:** Ibrahim Burak Ozyurt, Jeffrey S Grethe

**Affiliations:** Center for Research in Biological Systems, University of California, San Diego, La Jolla, CA, USA

## Abstract

Data generated by scientific research enables further advancement in science through reanalyses and pooling of data for novel analyses. With the increasing amounts of scientific data generated by biomedical research providing researchers with more data than they have ever had access to, finding the data matching the researchers' requirements continues to be a major challenge and will only grow more challenging as more data is produced and shared. In this paper, we introduce a horizontally scalable distributed extract-transform-load system to tackle scientific data aggregation, transformation and enhancement for scientific data discovery and retrieval. We also introduce a data transformation language for biomedical curators allowing for the transformation and combination of data/metadata from heterogeneous data sources. Applicability of the system for scientific data is illustrated in biomedical and earth science domains.

## Introduction

Modern biomedical science involves the accrual of increasingly larger data sets in many forms. SciCrunch (https://scicrunch.org) lists >2800 databases in its resource registry, spanning biological systems from the level of genes to behavior. We know that the amount of data that is easily discoverable and accessible relative to the amount produced or that which is required to comprehensively cover a domain is limited. Therefore, calls to add to public data must also be accompanied by platforms for making these data available, searchable and useful (FAIR, a set of guiding principles to make data Findable, Accessible, Interoperable, and Reusable) (1). These challenges are not unique to specific disciplines but echo the needs of science in general (2). Individual communities are also not unique in wanting to determine the most effective means to maximize the utility of existing resources created to support and spur their researchers. Our experience in Neuroscience Information Framework (NIF) and dkNET (3–7) suggests that researchers are largely unaware of the many resources that are available to them, not just targeted resources such as the Mouse Metabolic Phenotyping Center but also databases such as the National Institutes of Health (NIH) Reporter. The current funding situation requires that we take full advantage of the available resources to create effective platforms for data generation, sharing and analysis. Most recently, as part of the NIH Big Data to Knowledge initiative, the biomedical and healthCAre Data Discovery Index Ecosystem (bioCADDIE, RRID:SCR_004018) (8, 9) was launched to build a prototype (DataMed, http://datamed.org) for data discovery analogous to PubMed. In order to support these discovery needs, there is a need to develop and deploy technologies to make the large collection of data and information resources collectively searchable.

Development of the Foundry indexing infrastructure was informed by the following three distinct user communities: (i) biomedical data set discovery (bioCADDIE), (ii) biomedical information and discovery portals (NIF, RRID:SCR_002894 and dkNET, RRID:SCR_001606) and (iii) Earth science resource discovery [EarthCube Community Inventory of EarthCube Resources for Geosciences Interoperability (CINERGI), RRID:SCR_002188] (10). The umbrella system was designed to interoperate with the current ecosystem of indices and repositories and not to replace them. The overall infrastructure consists of the following components:

- A data and metadata extraction system that is able to connect to various repositories and data aggregators/integrators using parameterized ingestors and a domain-specific language (DSL) to specify complex data extraction/ingestion scenarios. Incoming metadata information is converted to JavaScript Object Notation (JSON) for each data set being described and is stored in MongoDB.
- A loosely coupled distributed data processing pipeline management system using a message-oriented middleware (MoM) architecture, utilizing Apache ActiveMQ (http://activemq.apache.org/), to manage a set of enhancements and transformations on the ingested data for data integration.
- A generic transformation language to align heterogeneous data from multiple sources with a data/metadata model [e.g. bioCADDIE Data Tag Suite (DATS) (9)] enabling a broader community of curators to create and manage the transformation of data and metadata.
- Export mechanisms for the enhanced/transformed data to Elasticsearch search engine endpoints or to local file structures.

### Related work

With the availability of large-scale data, the importance of extract-transform-load (ETL) systems to prepare data for analytics have increased. Processing of big data requires usage of new programming models such as MapReduce (11) on a cluster of machines. Hadoop is the most popular open-source implementation of MapReduce model acting as a generic ETL framework allowing programmers to write code for their specific ETL task. Apache Spark addresses some of the shortcomings of Hadoop for iterative large-scale data processing and interactive data analytics. However, Apache Spark is also a low-level ETL framework designed for programmers. Foundry, on the other hand, is a distributed ETL system specifically designed for scientific data with declarative ingestion and transformation languages for data curators.

Foundry is most similar to the Discovery (DISCO) system (12, 13) (RRID:SCR_004586) currently used by SciCrunch.org for data ingestion and integration. While Foundry has a similar end goal as DISCO of being an ETL system for scientific data harvesting and integration, Foundry differs from DISCO in many aspects. Foundry is designed to be a loosely coupled non-monolithic cloud-based ETL system that can scale with additional data load horizontally by adding new consumer containers running on additional machines to distribute the processing load. It uses streaming iterators for handling very large data sets and has an ingestion language for complex data ingestion pipeline construction. It also has a generic transformation language for transforming, cleaning and normalizing data records. Another major difference from DISCO is workflow management for executing a configured set of discrete processes to enhance the data records.

## Materials and methods

### System design

MoM systems such as Apache ActiveMQ allow designing loosely coupled distributed systems that can run in heterogeneous distributed computer environments. Use of MoM in Foundry allows horizontal scaling of the system by adding new consumer nodes on demand for increased load. It allows for event-driven, reactive workflow management orchestrated by messages sent/received from consumers that are self-contained processing units (microservices) to/from a message dispatcher component. The persistent message queues also make the system resilient to computer crashes and outages, as unfinished messages resulting from a computer failure on a consumer node remain in the message queue and are either picked up by another consumer node (if any are running) or reprocessed when the consumer node restarts.

**Figure 1:**
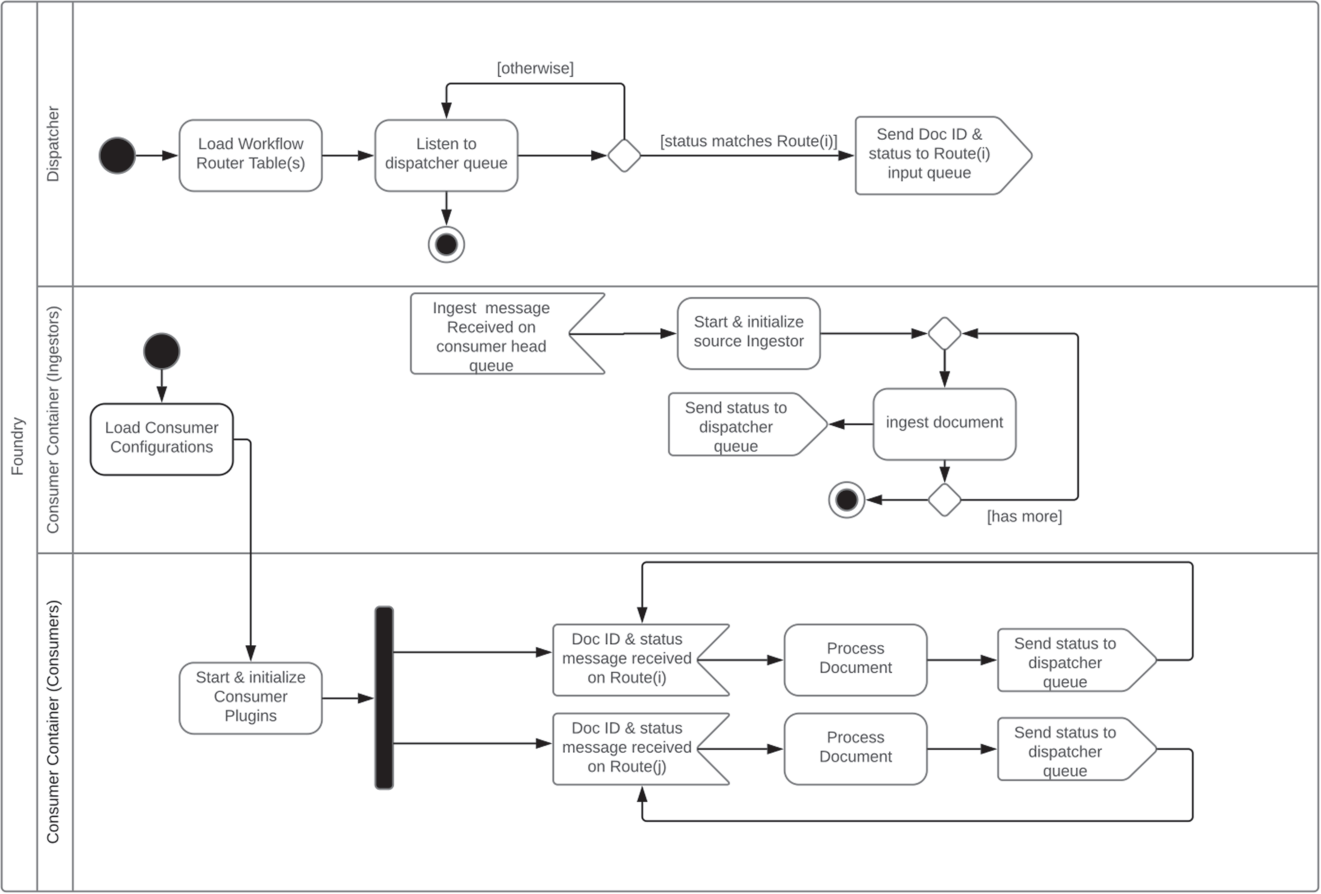
Foundry dispatcher and consumer container unified modeling language activity diagram.

The overall system consists of a dispatcher, one or more consumer container(s) and a command-line manager interface. The dispatcher listens to the dispatcher message queue for incoming messages from consumer container instance(s). Using its configured workflow, it dispatches messages to the message queue for the listening consumer container(s). The consumer container coordinates a set of configured consumers that perform predefined operation(s) of a document indicated by the message they receive from the dispatcher and ingestors. The harvesters/ingestors are specialized consumers that are responsible for the retrieval of the original data as configured by a harvest descriptor JSON file of the corresponding source. They are triggered by the manager application. These component interactions are summarized in Figure 1 and an example of a specific pipeline is given in the section below.

### Dispatcher architecture

The dispatcher subsystem coordinates messages coming from consumers/enhancers and relays them to corresponding message queues as configured by a particular data enhancement/transformation workflow. Each workflow is configured as a routing table. A route associates a status label with a message queue. Special status labels are specified to indicate the start and the end of the pipeline. Each consumer/enhancer is configured to listen for messages with a certain status label on a particular message queue. When the consumer finishes processing a data record, it puts a message to a preconfigured message queue with a consumer-specific output status label for the dispatcher to pick up. The dispatcher creates a new message with the output status label received and puts it to the message queue of the matching route in the pipeline route table. This process continues until there is no route left in the pipeline route table. The dispatcher is a lightweight component acting as an event bus. The heavy lifting is done by the consumers running inside the consumer container(s).

**Figure 2:**
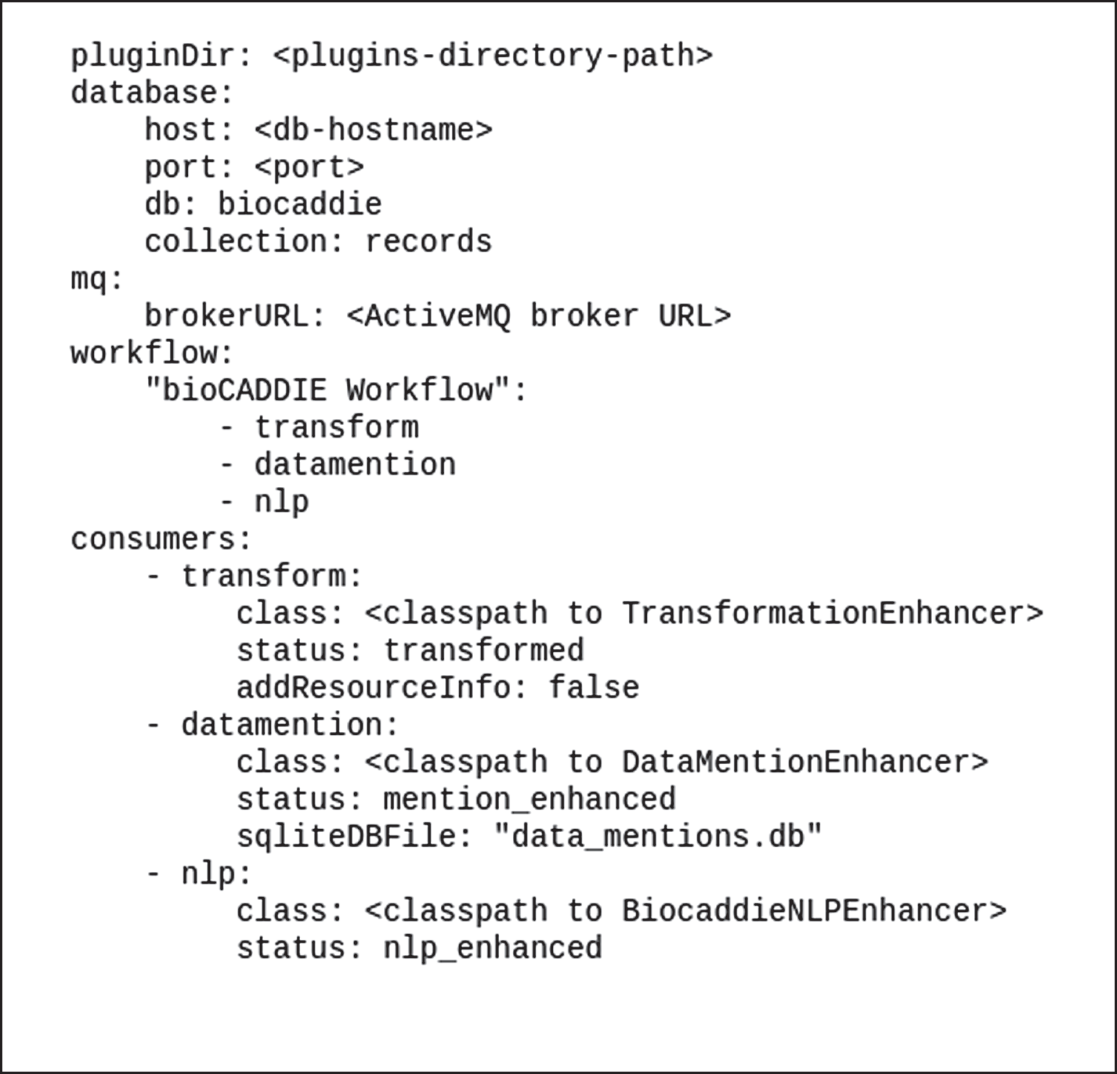
Meta configuration file for the Foundry system.

The whole system including the workflow and the enhancers is configured from a single configuration file. For example, the bioCADDIE data processing workflow consist of a transformation that aligns the metadata to the DATS format (9), citation enhancement (datamention) and biomedical named entity detection enhancement [natural language processing (NLP)] steps as illustrated by the YAML Ain't Markup Language (YAML, a human-readable data serialization language) configuration file used for bioCADDIE in Figure 2. The configuration file has four main sections: a database section for Mongo database configuration, a message queue (mq) section for message queue connection configuration, a workflow section to specify the pipeline as a list of consumer aliases and a consumers section to configure individual consumers/enhancers used by the pipeline. Each consumer configuration has two mandatory fields, namely, ‘class’ to specify the full java class name of the consumer implementation and ‘status’ indicating the status of the processed record after this consumer has finished processing it. The status label is used to name the internal message queues used to orchestrate the pipeline processing and should be unique for each consumer. Any additional options specific to a particular consumer are provided as name–value pairs besides mandatory class and status fields.

### Consumer container architecture

The consumer container coordinates a set of consumers. A consumer listens to a preconfigured message queue and applies a predefined operation to the document indicated by the received message. On success, a consumer sets the processing status of the document to a preconfigured status. Consumers are implemented as plugins with life cycle methods that are called by the consumer container during the life of the consumer in the container. The life cycle events include the creation, initialization and shutdown of a consumer. The Foundry framework provides support for two types of consumers, namely, ingestors and enhancers. The ingestors are responsible for the extraction/retrieval of data/metadata from a data source. The enhancers are responsible for transforming and enhancing the extracted source data. Each consumer runs in its own thread. The consumer container spawns only one consumer instance for each enhancer type and spawns one ingestor per data extraction source. An ingestor stays alive until all the data records from that source are extracted and terminated by the container afterwards. Enhancers are only terminated when the consumer container terminates. The consumer container is also responsible for duplicate checking and document wrapper generation.

#### Harvesters (ingestors)

All ingestors are implemented as plugins. The Ingestor interface has life cycle methods to initialize parameters received in the message body to start the ingestion process. These include the harvest url and ingestion type-specific parameters defined in the harvest description JSON file stored in the MongoDB under the sources collection. The startup() life cycle method is generally used to get the data to an intermediate storage/cache. An ingestor plugin acts like an iterator where the hasNext() method returns true if there are still more records to process and the prepPayload() method returns a JSON representation of the original record harvested.

The harvesters form the extract portion of the Foundry ETL system. Both the distribution and format of the scientific data are heterogeneous. Hence, both access mode and data format need to be taken into account in devising a generic design for the harvester portion of the system. The generalizable parts, namely, the access mode of the raw data [e.g. via FTP, RSync, web application programming interface (API) or a file bundle] and distribution format (e.g. XML, CSV or JSON) are abstracted out. All available harvesters are parametrized to increase reusability. Another responsibility of a harvester is partitioning of the data into records to iterate over. The harvesting framework relies heavily on the iterator design pattern (14) similar to cursors used in relational database systems. In relational database systems, structured query language (SQL) queries are converted to a pipeline of relational operations on a set of cursors for the tables and indices involved in the query during the query planning phase (15). To support data sets that will not fit the system memory, the devised iterators are lazy and retrieve the next record on a demand basis in a streaming fashion allowing the system to process very large data files such as 0.5 TB XML file dumps from UniProt (16) (RRID:SCR_002380). The harvester support system has iterators for the scientific data distribution formats we have encountered so far. The types of ingestors used for the bioCADDIE project are summarized in Table 1.

**Table 1:** Ingestors/Harvesters used for bioCADDIE

**Ingestor type**	**Sample bioCADDIE sources**	**Number of sources using this ingestor**
Web service ingestor	Clinical Trials, Uniprot	42
Database ingestor	NeuroMorpho, PeptideAtlas, Clinical Trials Network	14
OAI-PMH ingestor ^†^	Dryad, The Cardiovascular Research Grid	2
Two-stage web service ingestor ^*^	Inter-university Consortium for Political and Social Research, Dataverse Native	2
Rsync ingestor	PDB, dbGAP	2
FTP ingestor	BioProject, Biological Magnetic Resonance Data Bank (BMRB)	2
Aspera ingestor	GEO Datasets	1
CSV ingestor	Gemma	1
XML ingestor	ArrayExpress	1

The web service ingestor uses REST API of the source to access raw data. ^*^The two-stage web service ingestor also uses REST API; however, the data is accessed in two steps: first, finding the identifiers of the available data, usually through a web service providing search or summary listings, and then by retrieving each data record, through a more detailed service, by the extracted identifiers. ^†^The Open Archives Initiative Protocol for Metadata Harvesting (OAI-PMH) ingestor implements OAI-PMH protocol to retrieve archived data.

#### DSL for data ingestion

For some of the data sources, an extraction step is involved, including multiple interdependent steps to access data records and/or joining multiple data file/API call results to form the data record to be extracted. To this end, we have developed a DSL for data extraction/ingestion available as a generic ingestor/harvester. Similar to the way a database management system handles the query planning and processing, the harvester language interpreter converts the declarative instructions into a set of iterators (cursors) that can be joined together after determining the join order.

##### Ingestion DSL syntax

**Figure 3:**
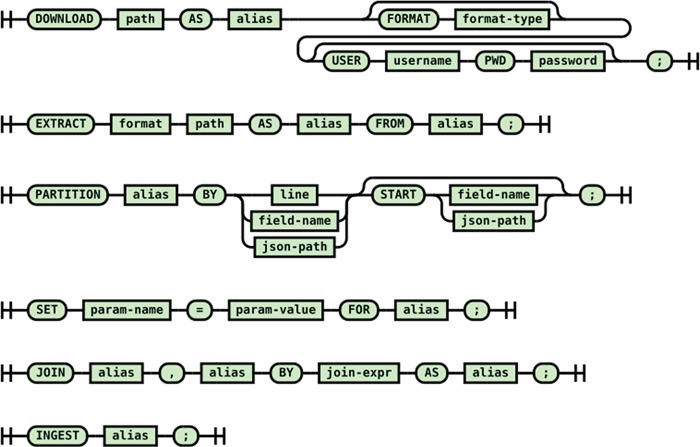
Syntax diagram of the DSL for the retrieval/combining/ingestion of the raw scientific data.

The syntax of the ingestion DSL is shown in Figure 3. For all common harvesting operations such as downloading the raw data, extracting files from data distribution bundles, partitioning the data files into individual records and joining multiple raw records into a record to ingest, a DSL statement is provided. The statements in an ingestion script form a pipeline of low-level operations to prepare raw data for ingestion into the Foundry system. Statements such as ‘DOWNLOAD’ and ‘EXTRACT’ generate artifacts for the data processing statements such as PARTITION and JOIN which are identified by aliases. The aliases are used in upstream statements to identify artifacts for partitioning and combining. The script ends with an INGEST statement. From the declarative ingestion DSL script, the ingestion DSL engine creates an ingestion data preparation pipeline using the built-in cursors/iterators of the Foundry framework. The cursors can be parametrized using the SET statement as needed.

**Figure 4:**
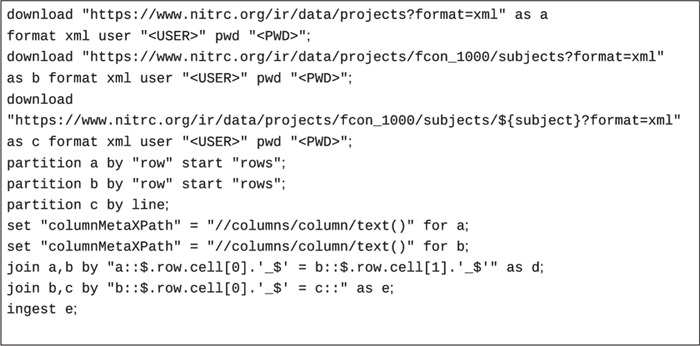
A sample ingestion script to retrieve and join data from three web services to form self-contained metadata records to be ingested.

An example script to extract records from the NITRC-IR data resource (17) (RRID:SCR_004162) by joining project, subject and subject data information are shown in Figure 4.

Here, raw data is retrieved from three representational state transfer (REST) API calls (the last call is parametrized) in XML format and cached locally. Since the data retrieved contains multiple data records, they are partitioned into records via ‘partition’ statements. Each ‘row’ tag in the first two XML documents under the ‘rows’ XML tag indicate a data record. The last data set is parametrized by the subject identifier and contains only one record. It is retrieved on demand by the three-way inner join indicated by the ‘join’ statements. The fields to join on are declared as JSONPaths, since all row data formats are converted to JSON first for internal processing. The value for each parameterized REST API retrieval call comes from the value of the join data record on the left-hand side of the join. The data record to be ingested is a combination of data records from the project, project subjects and individual subject information joined by the common (join) fields indicated in the join statements in Figure 4.

#### Consumers/enhancers

The consumer container subsystem of the Foundry framework manages the lifecycle of the consumers, the main units of work, of the system. Each consumer is responsible for a single atomic process applied to a data record. They are stateless and work concurrently on different data records. However, on the same record the order of operations is determined by the configured data/document processing pipeline orchestrated by the dispatcher. Consumers are implemented as plugins to a generic consumer process to simplify third party consumer development and to facilitate customizability and extensibility of the system. Transformation and data enhancement are the most common usages for consumers. Each consumer listens on a predefined message queue for messages from the dispatcher. These messages consist of the current status and the id of the data record with which the wrapper for the data record is retrieved from the MongoDB database, processed and written back before signaling the dispatcher about the current status of the document. The status messages are used by the dispatcher to determine the next consumer in the pipeline.

The types of document enhancements needed are application specific. Thus, different sets of enhancers have been developed for the projects where Foundry is used. For bioCADDIE, an NLP pipeline was developed by members of the bioCADDIE Core Development Team at the University of Texas Health Science Center at Houston for recognizing gene, disease, drug and cell line mentions in the data record metadata descriptions. This code module was converted to an enhancer using the client side plugin API. To incorporate literature citation information for data records from Protein Data Bank (PDB) (18) (RRID:SCR_012820), GEO (19) (RRID:SCR_005012) and other data sets, another enhancer was also developed that incorporates citation information from an external web service.

Since the CINERGI project is focused on enhancement of geological metadata records, time was devoted to developing an ontology-backed NLP enhancer for keyword extraction and suggestion from the metadata record, a geolocation enhancer with location named entity recognition and an organization enhancer to associate organizations in free text format in the geological metadata records with their corresponding Virtual International Authority File records.

### Data transformation language

Aggregation of heterogeneous semi-structured data from multiple sources requires alignment and transformation to a common format. To this end, we have developed a simple, curator friendly, transformation language to transform a given hierarchical data structure in JSON format to any desired JSON form including JSON-LD for linked data.

The first thing that needs to be addressed is how to indicate a path(s) in a JSON tree. For XML, there is the XPath specification to address this issue. However, there is no parallel standard for JSON. However, by analogy to the XPath specification there are attempts to implement/specify paths in a JSON tree. We have developed a subset of the analogous functionality to XPath as JSONPath based on the syntax from https://goessner.net/articles/JsonPath to be used in the JSON transformation language (JSONTL). Our JSONPath implementation is tightly integrated with our transformation engine to allow complex nested multivalued source JSON forest to destination JSON forest transformations besides speed optimizations. The transformation language named JSONTL allows declaration of a mapping from a source path to a destination path optionally combining and/or transforming the source value. To support arbitrary value transformation, the JSONTL integrates the Python programming language. This way, common operations such as data cleanup, conversion, normalization, combining multiple fields, splitting values and generating calculated fields based on existing ones are made possible.

#### JSONPath syntax

**Figure 5:**
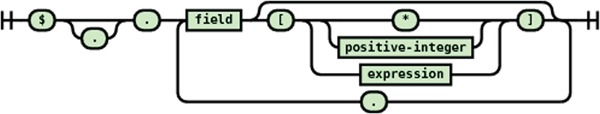
JSONPath syntax for the source document.

**Figure 6:**
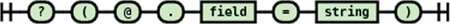
JSONPath expression syntax recognized.

**Figure 7:**
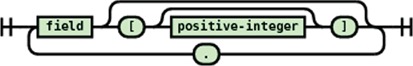
JSONPath syntax for the generated target JSON document.

For the source side of a transformation statement, the JSONPath is used to match path(s) and for the destination side, the JSONPath is used to create a new path (branch in the JSON document tree). Due to this functionality difference, the recognized syntax of the source and destination JSONPaths are slightly different. The syntax diagrams for source document JSONPath syntax are shown in Figures 5 and 6, the target document JSONPath syntax is shown in Figure 7.

An example of JSONPath for a source document, taken from the transformation script for the PDB, is ‘$..'PDBx:database_PDB_revCategory'.'PDBx:database_PDB_rev'[?(@.'@num' = '1')].'PDBx:date'.'_$'’. This statement matches the text (‘$\_\$$’) of the PDBx:date object in the PDBx:database_PDB_rev object having a field named ‘@num’ (mapped from the ‘num’ XML attribute) with value equal to 1. The ‘$..’ at the beginning of the statement indicates that the remaining subtree is matched at an arbitrarily deep level in the source document JSON hierarchy.

An example of JSONPath for a destination document from the transformation script for PDB is ‘identifiers[].ID’ where the destination document will have an array called ‘identifiers’ at the top level having objects with a field named ‘ID’.

#### JSONTL syntax

The transformation language consists of five types of statements allowing one-to-one, one-to-many, many-to-one and many-to-many mappings of various sorts. Besides the join statement, all JSONTL statements can be conditioned based on value, existence and non-existence of any source document field, using the optional conditional expression.

#### Constant field generation

**Figure 8:**
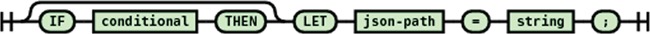
Syntax diagram for constant field transformation statement.

This statement (see Figure 8) allows introducing fields with constant values that does not exist in the source record. This statement is mostly used to add metadata about the data source to processed records.

An example constant field statement from the PDB transformation script is let "dataRepository.name" = "Protein Data Bank";

This statement assigns the value ‘Protein Data Bank’ to the ‘name’ field of a top level destination object named ‘dataRepository’.

#### Single path transformation

**Figure 9:**
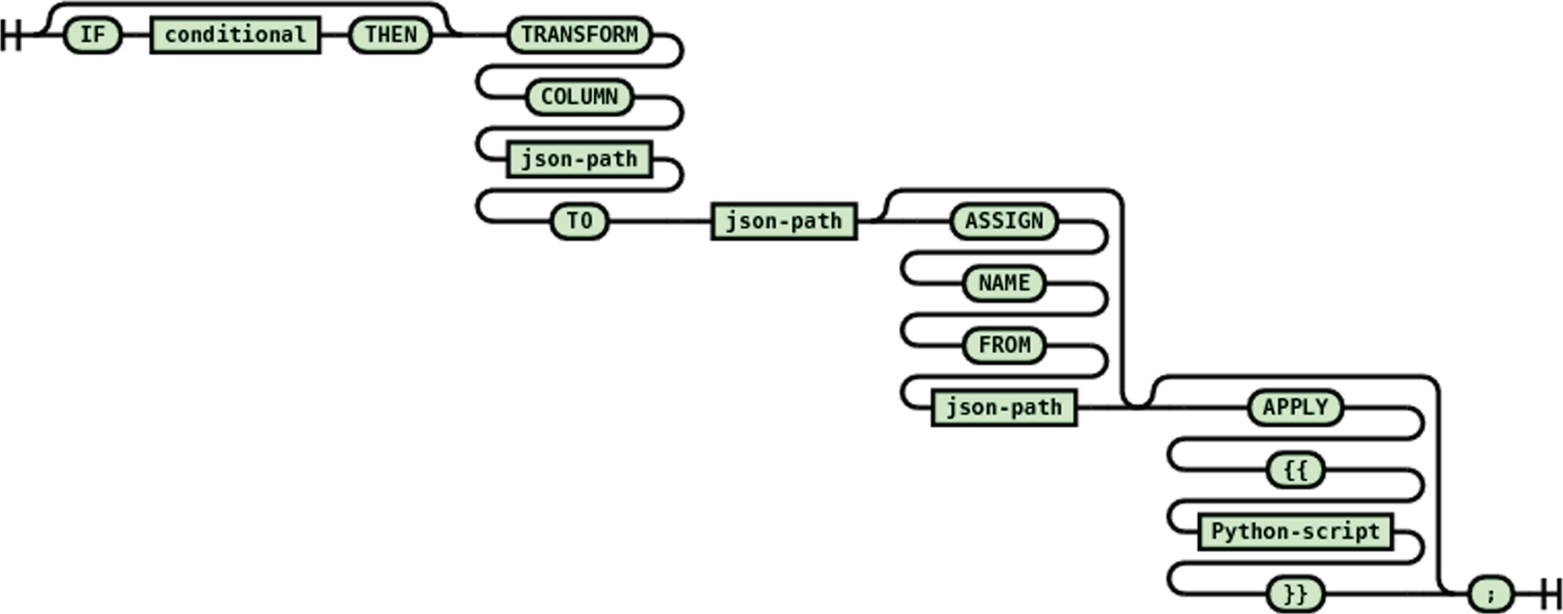
Syntax diagram for single path transformation statement.

Single path transformation (see Figure 9) allows transforming of a single source path to a single destination path with single value (one-to-one transformation) or multiple values (one-to-many transformation). One-to-many transformations are achieved by using the optional apply block that allows arbitrary manipulation of the matched source path value such as splitting delimited text into a list of keywords.

An example from the PDB transformation script with an apply block shows creating a landing page URL using the entry id value from a PDB record.

transform column "$.'PDBx:datablock'.'@datablockName'.'PDBx:atom_sites'.'@entry_id'" to "access.landingPage" apply {{ result =' http://www.rcsb.org/pdb/explore/explore.do?structureId= ' + value}};

Usage of built-in date processing functions with NLP capabilities for heterogeneous free form date fields can be illustrated by the following example from the PDB transformation script.

transform column "$..'PDBx:database_PDB_revCategory'.'PDBx:database_PDB_rev'[?(@.'@num' = '1')].'PDBx:date'.'_$'" to "dataset.dateReleased" apply toStandardDateTime("yyyy-MM-dd");

The transformation language is extensible with new functions that can be used instead of the APPLY Python script block as shown in the above example. The ‘toStandardDateTime()’ function used above is provided by implementing the transformation language’s plugin interface and registering it with the transformation engine. This function is used for converting date fields to internal date and time format suitable for Elasticsearch indexing and also allows free form date processing using NLP.

**Figure 10:**
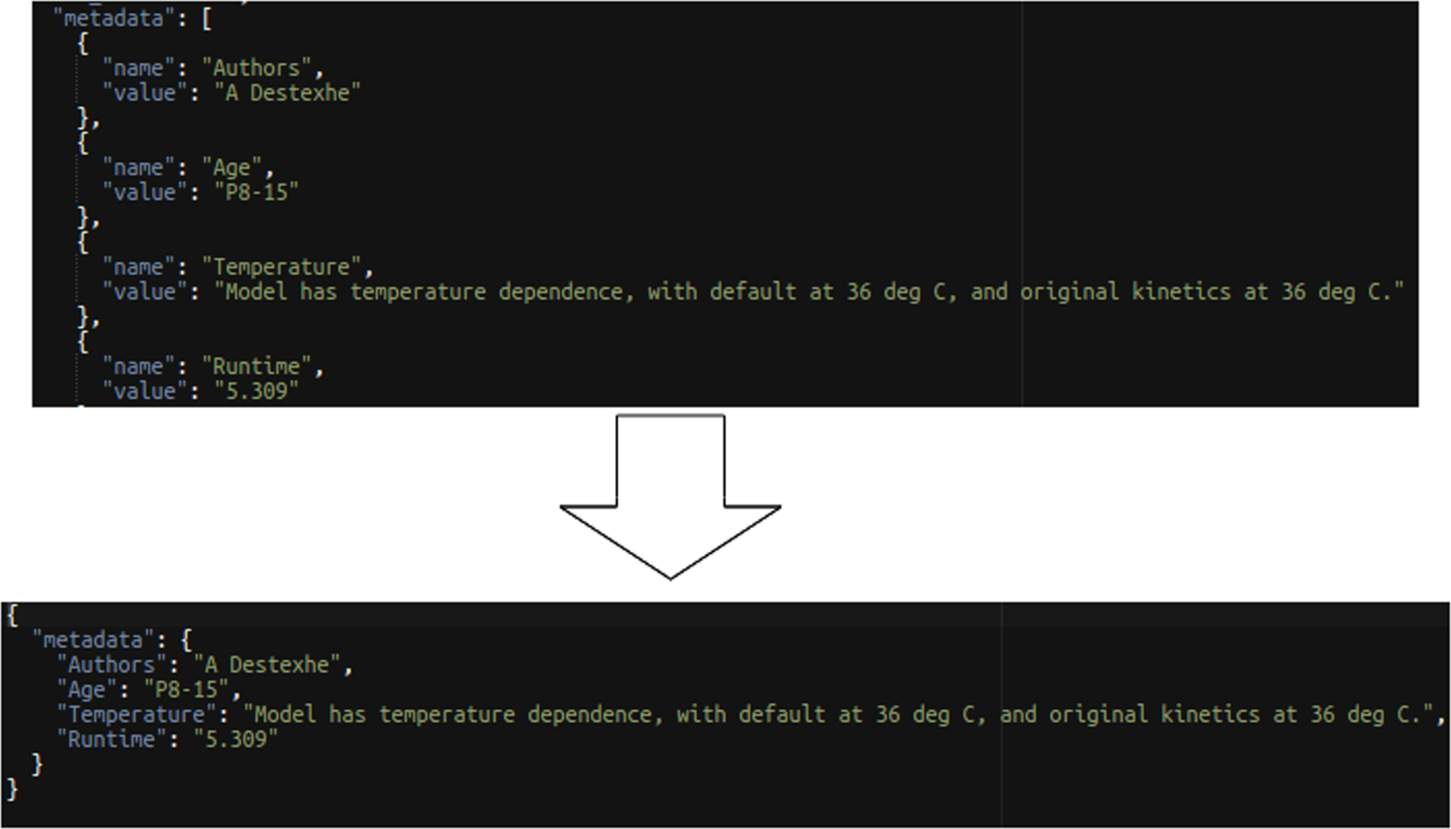
EVA style data transformation example.

The entity–value–attribute (EVA) model is a common extensible data model where data fields are encoded as name–value pairs allowing different data records to contain different set of data fields. To enable transformation of EVA-style data records, an ASSIGN NAME FROM construct is provided. The usage of an EVA-style transformation from the Ion Channel Genealogy transformation script is shown below:

transform column "$.'metadata'[*].'value'" to "metadata.value" assign name from "$.'metadata'[*].'name'";

This statement converts each name and value field pair from the source into a single field named from the source name field with the value of the source value field as demonstrated in Figure 10.

#### Multiple path transformation (many to one, many to many)

**Figure 11:**
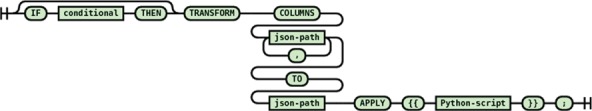
Syntax diagram for the multiple path many-to-one transformation statement.

This transformation statement (see Figure 11) is similar to single path transformation statements in structure. However, it allows for the combining of values from multiple source paths to a destination path through the help of the mandatory APPLY section.

The following statement from the PDB transform script illustrates how this statement is used in practice.

transform columns "$..'PDBx:struct_keywords'.'PDBx:pdbx_keywords'.'_$'", "$..'PDBx:struct_keywords'.'PDBx:text'.'_$'" to "dataset.keywords[]" apply {{

arr=re.split("\s*,\s*",value1,)

arr.extend(re.split("\s*,\s*",value2))

result=arr

}};

Here a multiple comma separated lists of keywords in the source document are combined into a single array of keywords after splitting the source keyword text fields into individual keywords via the Python code in the apply block of the statement.

#### Multiple path union transformation

**Figure 12:**
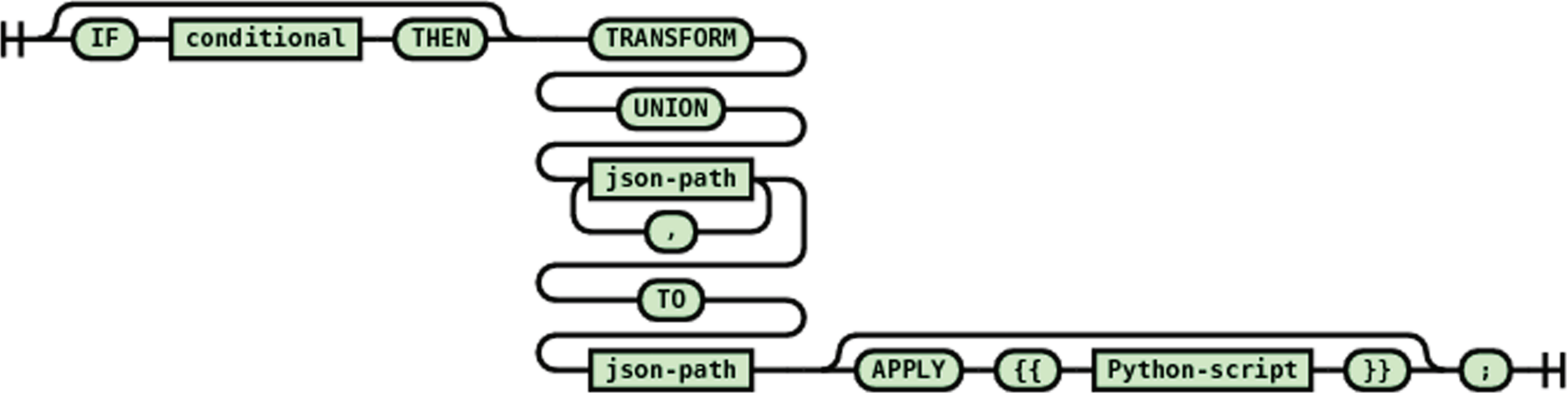
Syntax diagram for the multiple path union transformation statement.

This transformation statement (see Figure 12) combines all values from all the matching source paths and passes them all together to the destination path that needs to be multivalued (i.e. array). Each source value becomes another element in the destination array. Each source value can be processed by an optional apply block before being assigned to the destination element.

The following statement from the PDB transformation script illustrates a practical usage of the multiple path union transformation statement:

transform union "$.'PDBx:datablock'.'PDBx:entity_src_genCategory'.'PDBx:entity_src_gen'[*].'PDBx:pdbx_gene_src_ncbi_taxonomy_id'.'_$'", "$.'PDBx:datablock'.'PDBx:entity_src_genCategory'.'PDBx:entity_src_gen'[*].'PDBx:pdbx_host_org_ncbi_taxonomy_id'.'_$'" to "taxonomicInformation[].ID" apply {{ result = 'ncbitax:' + value }};

Here the taxonomy identifiers from the source and host organisms are combined together and passed one by one into the apply block to generate a CURIE (https://www.w3.org/TR/curie/), an abbreviated syntax for expressing uniform resource identifiers, for the passed taxonomy identifier.

#### Join transformation (many to one, many to many)

**Figure 13:**
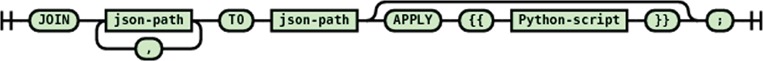
Syntax diagram for the join transformation statement.

This transformation statement (see Figure 13) passes a list of values of each matching source path forest, as identified by its JSON Path, to the optional APPLY block. If no apply block is specified, all values from the source are combined to a comma separated list that becomes the value of the destination path. However, the power of this statement is realized with an apply block allowing, for example, one to select a corresponding source value based on the value(s) of another field’s list.

An example usage of this statement from the UniProt-SwissProt BioCADDIE transformation script is show below:

join "$.'entry'.'organism'.'name'[*].'@type'", "$.'entry'.'organism'.'name'[*].'_$'" to "taxonomicInformation[0].name" apply

{{

i = -1

try:

i = value1.index('scientific')

except:

pass

result = value2[i] if i >= 0 else “

}};

This statement takes in a list of organism names and a list of organism name types, identifies the organism name with the type ‘scientific’ and assigns the found scientific organism name to the destination as the taxonomic information name.

#### Conditional expression

**Figure 14:**
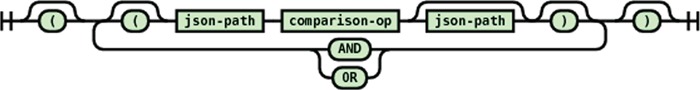
Syntax diagram for the conditional expression supported in JSONTL.

**Figure 15:**
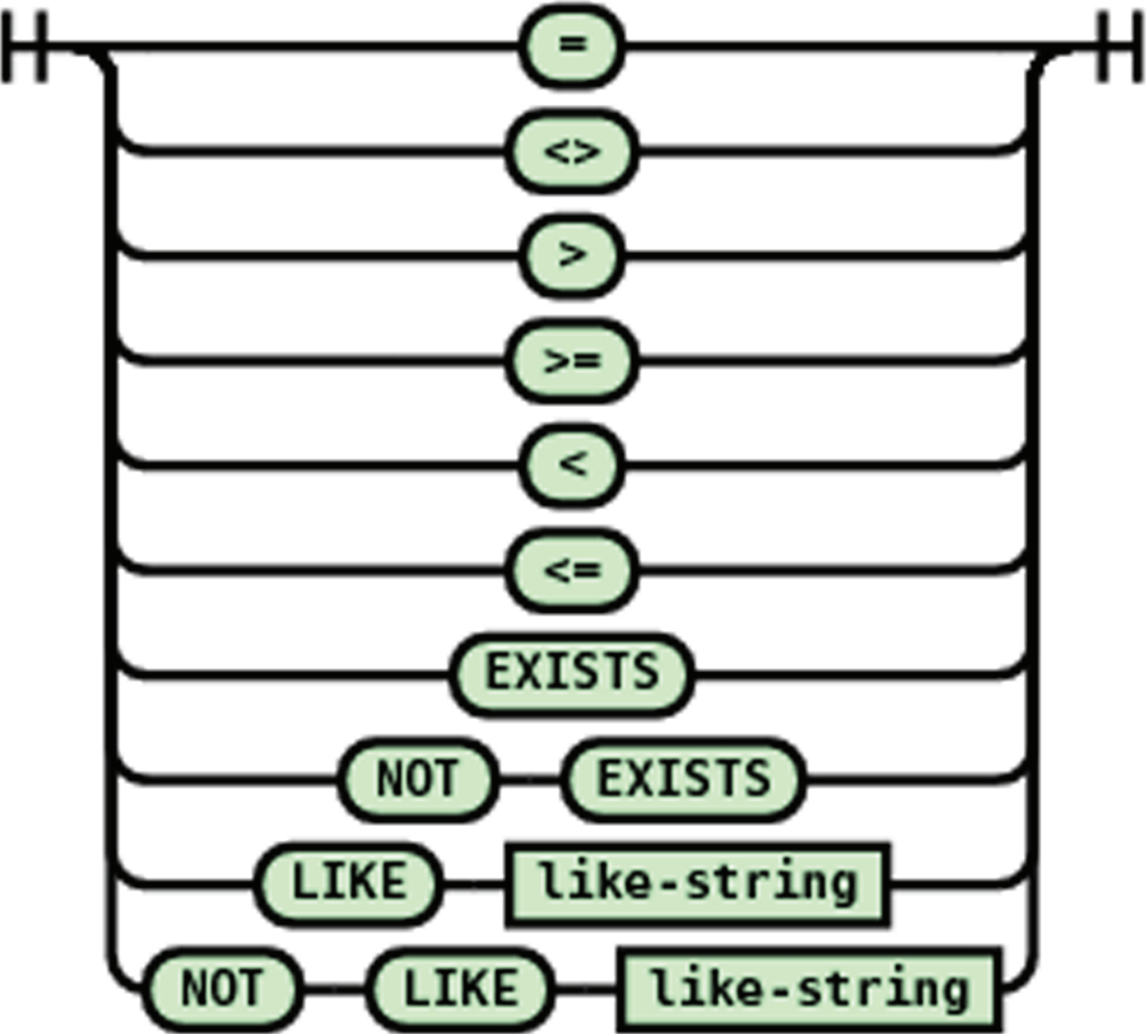
Syntax diagram for the conditional operators supported in JSONTL.

JSONTL supports conditioning of a transformation statement on arbitrary number of source path values by a wide array of comparison operators. The syntax diagrams of the conditional expression and operators are shown in Figures 14 and 15.

A practical example of conditional expression usage from the transformation script for Gemma (20) (RRID:SCR_008007) is shown below:

if "$.'SourceAccession'" like "%GSE%" then let "datasetDistributions[2].storedIn" = "Gene Expression Omnibus";

Here if the ‘SourceAccession’ field value contains the string ‘GSE’, then a constant field is generated on the destination.

### Data export

The ability to export transformed and/or enhanced data is essential for an ETL system. The exported data is used by the upstream systems. In the case of bioCADDIE, the enhanced data is indexed to an Elasticsearch endpoint used by the DataMed UI. In the case of the CINERGI project, the enhanced ISO XML metadata documents are exported as files for indexing within an ESRI Geoportal server (https://www.esri.com/en-us/arcgis/products/geoportal-server/overview). The export functionality is implemented as another enhancer/consumer in the Foundry system configured to be the final step in the processing pipeline.

## Results and discussion

The introduced transformation language enables incremental development of data transformations since each transformation rule only handles a small number of branches (usually only one branch) in the source data hierarchy. Thus, the rules can be developed and tested individually. A web front-end for testing the transformation rules is part of the system to facilitate incremental development. In DISCO, transformation and data aggregation of a data source is done in a single SQL query that usually ends up being overly complex making it hard to maintain requiring expert level SQL skills. Also, the transformations are limited to the capabilities of SQL in a single large query. With its embedded Python programming language execution capabilities, the introduced transformation language provides data manipulation capabilities of a full-blown computer language. The system includes a tool to automatically generate identity transformation rules from raw sample data as an aid to the curators to minimize effort. These features, together with the relative simplicity of the transformation language, lower the bar of entry for curators, decreasing data source transformation development and maintenance time. The transformation language was designed in concert with curators working on biomedical data curation for almost a decade in SciCrunch and with the input from BioCADDIE curators.

In Foundry, in contrast to DISCO, a raw data set is stored as a denormalized and potentially hierarchical document instead of a set of normalized relational database tables. This allows each data set to be self-contained without requiring costly join operations to access related data. One of the main design goals for Foundry is to push as many of the semantic enhancements to the offline from the online search processes to improve end user experience. Each self-contained data set can be semantically enhanced independent of each other in parallel and the additional semantic information is indexed together with the data/metadata enabling fast, semantically enhanced queries.

Foundry is open source and together with its documentation can be retrieved from the bioCADDIE GitHub repository (https://github.com/biocaddie/Foundry-ES).

**Figure 16:**
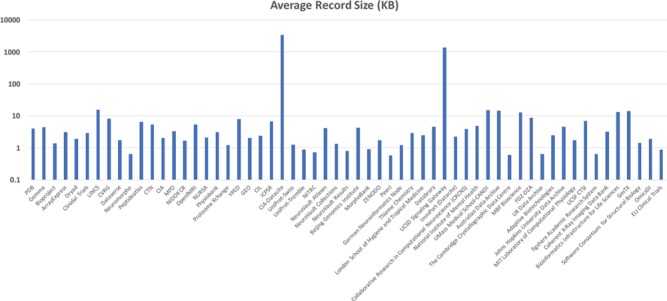
Average Metadata Record size across bioCADDIE DataMed test corpus.

Initial testing of the system was done on a system containing a single MongoDB instance running on an Amazon m4.large (8 GB RAM with two virtual CPUs) virtual machine and another m4.large virtual machine running the ActiveMQ server, Foundry dispatcher and a consumer container with ingestor enhancer. This testing was done with the initial bioCADDIE DataMed corpus consisting of 58 sources (77 085 123 metadata records) with a total size of 68.54 GB (median record size of 2.89 KB with a minimum size of 0.57 KB and a maximum size of 3.4 MB, see Figure 16).

Performance of the core system for metadata ingestion was measured for each source and took a total of 23 hours and 35 minutes (224 documents per second). Data transfer from the remote source was not included in the timings as this can be quite variable and is also determined by the remote sources policies for crawl rates and frequency. This base system performance can also be affected by the addition of various enhancement modules (e.g. NLP processing). In order to enable additional processing steps while maintaining throughput, the system needs to be scaled horizontally.

To test the horizontal scalability of the introduced system, we devised an experiment on a large data resource (full set of PubMED abstracts) on the Amazon cloud. The system consists of a single MongoDB instance running on an m4.large (8 GB RAM with two virtual CPUs) virtual machine, another m4.large running the ActiveMQ server, Foundry dispatcher and a consumer container with ingestor enhancer and four consumer containers running on four Amazon t2.medium (4 GB RAM with two virtual CPUs) EC2 nodes (virtual machines). Altogether, there are five computation nodes one of which is responsible for data ingestion and transformation and the other four only for transformation. For cloud deployment, we utilized two types of Amazon Machine Images (AMI), one for the node running the ActiveMQ server, dispatcher and the consumer container for ingestion and another for the slave nodes running consumer containers configured without the ingestion capability. The consumer AMI is then manually cloned to create the four consumer EC2 nodes. All the EC2 nodes are configured to be in the same zone and security domain. The consumer AMI can be used with the Amazon EC2 Auto Scale functionality to automatically start new consumer nodes on demand when load increases.

**Figure 17:**
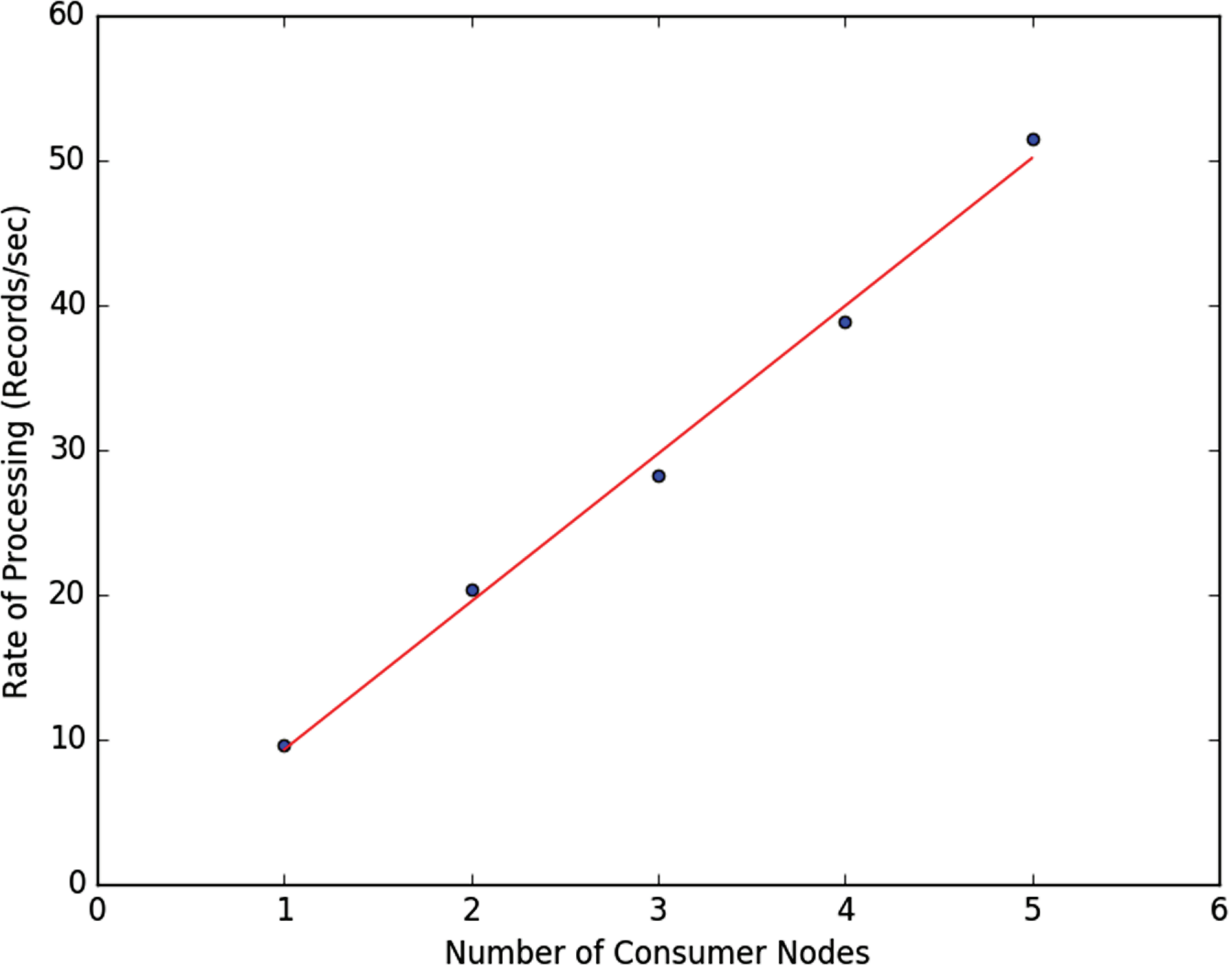
Horizontal scaling performance of the Foundry ETL system on the PubMed data ingestion and transformation.

We started with a master node and introduced each additional computation node one at a time while the pipeline is running. Each additional node is introduced at least one hour of processing apart and the rate of document processing after each introduced computation node is plotted together with the linear regression line in Figure 17, showing linear increase in processing rate. The Amazon EC2 nodes we have used are general purpose virtual machines with low to moderate network traffic settings using general purpose solid-state drives showing that decent data processing performance can be achieved even with run of the mill hardware with the introduced ETL system.

## Conclusion

In this paper, we introduced a scalable data integration/indexing platform that is being used across a wide variety of scientific domains. The system has been shown to have wide applicability across different scientific domains, metadata specifications and data types and formats. Through the transformation and enhancement process, a pipeline can be easily customized for a specific domain and associated use case. The transformation language and source configuration YAML files provide biomedical curators with an easy and understandable process to specify how a data source should be ingested. Current work is focused on development of additional enhancement modules that will support standardized mechanisms for query expansion and enhancement of links between indexed documents.

## Declarations

### Ethics approval and consent to participate

Not applicable.

### Consent for publication

Not applicable.

### Availability of data and materials

Foundry source code and documentation together is available on GitHub (https://github.com/biocaddie/Foundry-ES).

The data sets (i.e the indices) generated by the pipeline are available (and searchable) via the discovery portals for the projects involved in this work:

bioCADDIE: https://datamed.org

CINERGI: http://cinergi.sdsc.edu/geoportal/

Neuroscience Information Framework: https://neuinfo.org

NIDDK Information Network: https://dknet.org.

### Competing interests

I.B.O. has no competing interests.

Dr. Grethe has an equity interest in SciCrunch, Inc., a company that may potentially benefit from the research results. The terms of this arrangement have been reviewed and approved by the University of California, San Diego in accordance with its conflict of interest policies.

## Authors’ contributions

I.B.O. designed (together with J.S.G.) and implemented the introduced system and conducted the experiments on the system and was a major contributor in writing the manuscript. J.S.G. designed (together with I.B.O.) the system and implemented majority of the transformation scripts for bioCADDIE and was a major contributor in writing the manuscript. All authors read and approved the final manuscript.

## Notes

**Github URL**: https://github.com/biocaddie/Foundry-ES

## References

[1] Wilkinson M.D., Dumontier M., Aalbersberg I.J. et al. (2016) The FAIR Guiding Principles for scientific data management and stewardship. *Sci. Data*, 3, 160018. 26978244 10.1038/sdata.2016.18 PMC4792175

[2] Hey T., Tansley S. and Tolle K. (2009) *Fourth Paradigm: Data-intensive Scientific Discovery*. Microsoft Research, Redmond, Washington.

[3] Cachat J., Bandrowski A., Grethe J.S. et al. (2012) A survey of the neuroscience resource landscape: perspectives from the neuroscience information framework. *Int. Rev. Neurobiol.*, 103, 39–68. 23195120 10.1016/B978-0-12-388408-4.00003-4

[4] Gupta A., Bug W., Marenco L. et al. (2008) Federated access to heterogeneous information resources in the Neuroscience Information Framework (NIF). *Neuroinformatics*, 6, 205–217. 18958629 10.1007/s12021-008-9033-y PMC2689790

[5] Gardner D., Akil H., Ascoli G.A. et al. (2008) The neuroscience information framework: a data and knowledge environment for neuroscience. *Neuroinformatics*, 6, 149–160. 18946742 10.1007/s12021-008-9024-z PMC2661130

[6] Whetzel P.L., Grethe J.S., Banks D.E. et al. (2015) The NIDDK Information Network: a community portal for finding data, materials, and tools for researchers studying diabetes, digestive, and kidney diseases. *PLoS One*, 10, e0136206 10.1371/journal.pone.0136206. PMC4578941 26393351

[7] Bandrowski A., Brush M., Grethe J.S. et al. (2016) The Resource Identification Initiative: a cultural shift in publishing. *Brain Behav.*, 6, e00417. 10.1002/brb3.417 PMC4834942 27110440

[8] Ohno-Machado L., Sansone S.-A., Alter G. et al (2017) DataMed: finding useful data across multiple biomedical data repositories. *Nat. Gen.*, 49, 816–819. doi:10.1038/ng.3864. 10.1038/ng.3864 PMC6460922 28546571

[9] Sansone S.-A., Gonzalez-Beltran A., Rocca-Serra P. et al (2017) DATS: the data tag suite to enable discoverability of datasets. *Sci. Data*, 4, 170059. doi:10.1038/sdata.2017.59. 28585923 10.1038/sdata.2017.59 PMC5460592

[10] CINERGI, https://www.earthcube.org/group/cinergi (2017). (Accessed 15 May 2017).

[11] Dean J. and Ghemawat S. (2008) MapReduce. *Commun. ACM*, 51, 107.

[12] Marenco L.N., Wang R., Bandrowski A.E. et al. (2014) Extending the NIF DISCO framework to automate complex workflow: coordinating the harvest and integration of data from diverse neuroscience information resources. *Front. Neuroinform*., 8, 58. 25018728 10.3389/fninf.2014.00058 PMC4071641

[13] Marenco L., Wang R., Shepherd G.M. et al. (2010) The NIF DISCO framework: facilitating automated integration of neuroscience content on the web. *Neuroinformatics*, 8, 101–112. 20387131 10.1007/s12021-010-9068-8 PMC3819210

[14] Gamma E., Helm R., Johnson R. et al. (1994) *Design Patterns: Elements of Reusable Object-Oriented Software* (Adobe Reader). Pearson Education, Boston, MA.

[15] Garcia-Molina H., Ullman J.D. and Widom J. (2000) *Database System Implementation*. Prentice Hall, Upper Saddle River, New Jersey.

[16] UniProt Consortium (2009) The universal protein resource (UniProt). *Nucleic Acids Res.*, 37, D169–D174. doi:10.1093/nar/gkn664. 18836194 10.1093/nar/gkn664 PMC2686606

[17] Kennedy D.N., Haselgrove C., Riehl J. et al (2016) The NITRC image repository. *Neuroimage*, 124, 1069–1073, doi:10.1016/j.neuroimage.2015.05.074. 26044860 10.1016/j.neuroimage.2015.05.074 PMC4651733

[18] Berman H.M. (2000) The Protein Data Bank. *Nucleic Acids Res.*, 28, 235–242. 10592235 10.1093/nar/28.1.235 PMC102472

[19] Barrett T., Troup D.B., Wilhite S.E. et al. (2007) NCBI GEO: mining tens of millions of expression profiles—database and tools update. *Nucleic Acids Res.*, 35, D760–D765. 10.1093/nar/gkl887. 17099226 PMC1669752

[20] Zoubarev A., K.M., K.D. et al (2012). Gemma: a resource for the reuse, sharing and meta-analysis of expression profiling data. *Bioinformatics*, 28, 2272–2273. doi:10.1093/bioinformatics/bts430. 22782548 PMC3426847

